# Digital Health Applications: Toward a Lifecycle and Pay-for-Performance Approach

**DOI:** 10.1016/j.mcpdig.2023.07.001

**Published:** 2023-08-08

**Authors:** Johannes Knitza, Felix Muehlensiepen, Sebastian Kuhn

**Affiliations:** Department of Internal Medicine 3, Rheumatology and Immunology Friedrich-Alexander University Erlangen-Nürnberg and Universitätsklinikum Erlangen, Erlangen, Germany; Centre for Health Services Research Brandenburg, Brandenburg Medical School, Rüdersdorf, Germany; Institute of Digital Medicine, Philipps-University and University Hospital of Giessen and Marburg, Marburg, Germany

Digital health applications (DHAs) offer a versatile approach to overcome current medical care gaps. DHA are essentially mobile applications used for medical purposes. The omnipresence of smartphones promises to reach patients even in remote areas and provide continuous medical care in between face-to-face visits. DHA can be implemented along the entire patient pathway, starting with symptom checking, remote patient monitoring, and digital therapeutics (DTx). The number of DHA is continuously growing, and it is becoming increasingly difficult to identify high-quality DHA for health care professionals (HCPs) and patients alike.^1^

Successful clinical use of DHA is based on 4 interlocking pillars, as follows: (1) DHA development; (2) DHA evaluation; (3) DHA implementation; and (4) DHA education. To really improve a relevant care problem, early and close collaboration with patients and HCPs is essential. To ensure the best possible functionality and acceptance, early feasibility or usability studies are necessary. Qualitative research, including focus groups and interviews, complements classical quantitative research and enables in-depth feedback to improve DHA. To become a certified or even a prescription medical product, DHA needs to prove its safety and efficacy in clinical studies and postmarketing real-world studies. Organizational and health system-specific frameworks must be in place to implement DHA into standard care. Patients and HCPs need dedicated training to realize the full potential of DHA.

In this issue of Mayo Clinic proceedings: digital health Gilbert et al^2^ inform us about the purpose, regulations, different methods, and quality of DHA real-world performance monitoring. The authors stress the need for regular updates to DHA to continuously improve the quality and ensure safety and performance. Regulators increasingly understand that existing frameworks need to be adapted for DHA. The authors highlight that real-world evidence (RWE), on the basis of high-quality surveys, patient and clinician reported outcomes, is essential to enable a continuous DHA assessment.

We agree that the collection of RWE is crucial to continuously improve DHA quality and would like to draw the reader’s attention to the DHA lifecycle framework presented by Tarricone et al.^3^ Similarly, the authors pointed out the low adoption of DHA in clinical care and the lack of regulatory guidance. On the basis of a thorough review and focus group discussion, the authors formulated 10 recommendations. The framework stresses the early inclusion of end-users during DHA development and the measurement of patient empowerment associated with DHA use. We strongly agree and believe that patient empowerment and improved patient-doctor interaction are the main benefits of DHA usage. In line with Gilbert et al,^2^ the authors recommend including economic evaluations; however, also recommend evaluating the effect of DHA on equity with regard to the existing digital divide.^4^

European regulators are increasingly adapting to the use of RWE, profiting from longer experiences in the United States.^5^ In another highly recommended viewpoint, Stern et al^5^ share thoughts from roundtable discussions regarding a fast-track reimbursement and evaluation pathway for DHA in the German market. This accelerated pathway enables provisional reimbursement in a trial phase of 1 year (or in exceptions of up to 2 years) during which the manufacturer needs to complete a clinical study. In this study, the manufacturer needs to provide the necessary evidence to become permanently a prescription medical product.

Results of our recent German DHA pilot study^6^ highlighted poor adherence as a main and persistent DHA burden. Only 13% of the patients used the DHA regularly over 3 months. We believe that DHA and DTx particularly, are perfectly suited for pay-for-performance models linking RWE and reimbursement to limit unnecessary costs. A stepwise value-based approach could enable DTx reimbursement if the patient completed the DTx program, a stricter pay-for-performance approach would only enable reimbursement if the patient experienced a relevant benefit owing to DTx usage.

Hopefully the stimulating thoughts by Gilbert et al^2^ and others will prompt a rise in RWE collection, improved regulations, and overall implementation of DHA.

## Potential Competing Interests

Author JK declares Non-Financial Interests being part of the Digital Rheumatology Network steering board and the following Competing Financial Interests: he has or has had consulting relationships with ABATON, Abbvie, BMS, Böhringer Ingelheim, 10.13039/100010795Chugai, Galapagos, Gilead, GSK, Lilly, 10.13039/501100014841Medac, 10.13039/100004336Novartis, 10.13039/100011110UCB, Vila Health, Werfen and received study support from 10.13039/100006483Abbvie, ABATON, 10.13039/501100001659DFG, 10.13039/100014419EIT Health, 10.13039/100004336Novartis, 10.13039/100004339Sanofi, Thermo Fisher, UCB; Author FM declares that he received study support from 10.13039/100006483Abbvie and Novartis; Author SK is the founder and shareholder of MED digital.

## References

[1] Knitza J., Tascilar K., Messner E.M. (2019). German mobile apps in rheumatology: review and analysis using the mobile application rating scale (MARS). JMIR Mhealth Uhealth.

[2] Gilbert S., Pimenta A., Stratton-Powell A., Welzel C., Melvin T. (2023). Continuous Improvement of Digital Health Apps Linked to Real-World Performance Monitoring: Safe Moving Targets?. Mayo Clinic Proceeding.

[3] Tarricone R., Petracca F., Cucciniello M., Ciani O. (2022). Recommendations for developing a lifecycle, multidimensional assessment framework for mobile medical apps. Health Econ.

[4] Knitza J., Simon D., Lambrecht A. (2020). Mobile health usage, preferences, barriers, and ehealth literacy in rheumatology: patient survey study. JMIR mHealth uHealth.

[5] Stern A.D., Brönneke J., Debatin J.F. (2022). Advancing digital health applications: priorities for innovation in real-world evidence generation. Lancet Digit Health.

[6] Labinsky H., Gupta L., Raimondo M.G., Schett G., Knitza J. (2023). Real-world usage of digital health applications (DiGA) in rheumatology: results from a German patient survey. Rheumatol Int.

